# Multi-Objective Optimizations of a Serpentine Micromixer with Crossing Channels at Low and High Reynolds Numbers

**DOI:** 10.3390/mi9030110

**Published:** 2018-03-04

**Authors:** Wasim Raza, Sang-Bum Ma, Kwang-Yong Kim

**Affiliations:** Department of Mechanical Engineering, Inha University, Incheon 22212, Korea; wasimkr@live.in (W.R.); msb927@inha.edu (S-B.M.)

**Keywords:** micromixer, multi-objective optimization, Reynolds number, Navier-Stokes equations, surrogate modeling, pressure drop

## Abstract

In order to maximize the mixing performance of a micromixer with an integrated three-dimensional serpentine and split-and-recombination configuration, multi-objective optimizations were performed at two different Reynolds numbers, 1 and 120, based on numerical simulation. Numerical analyses of fluid flow and mixing in the micromixer were performed using three-dimensional Navier-Stokes equations and convection-diffusion equation. Three dimensionless design variables that were related to the geometry of the micromixer were selected as design variables for optimization. Mixing index at the exit and pressure drop through the micromixer were employed as two objective functions. A parametric study was carried out to explore the effects of the design variables on the objective functions. Latin hypercube sampling method as a design-of-experiment technique has been used to select design points in the design space. Surrogate modeling of the objective functions was performed by using radial basis neural network. Concave Pareto-optimal curves comprising of Pareto-optimal solutions that represents the trade-off between the objective functions were obtained using a multi-objective genetic algorithm at *Re* = 1 and 120. Through the optimizations, maximum enhancements of 18.8% and 6.0% in mixing index were achieved at *Re* = 1 and 120, respectively.

## 1. Introduction

Microfluidics is related to an expeditiously emerging technology enabling manipulation and control of minute volumes of fluids with high accuracy in a miniaturized system for various fluidic functions, such as transporting, metering, valving, mixing, reacting, and separating [1,2]. Micromixer is an integral component of the microfluidic systems that have promising impact in the fields of biomedical diagnostics, drug development, and chemical industry [3,4]. Efficient mixing of liquid samples is a challenging task for successful operation of different processes in the microfluidic systems. The flow nature in the microfluidic systems is laminar, due to low Reynolds number. Thus, the mixing of fluid species depends mainly on mass diffusion in the absence of turbulence. However, the diffusion-dependent mixing is relatively slow and ineffective. In order to enhance the mixing performance, numerous methods have been proposed during the last two decades [3,5,6,7].

Depending upon the working principle, micromixers are categorized either as a passive or as an active type. Active micromixers employ external energy sources, such as electrokinetic, ultrasonic vibration and magnetic field to generate flow perturbations inside the microchannel. Although active micromixers generally show excellent mixing capability and control during the mixing, high fabrication cost, and difficulty in integration with microfluidic systems make them less practical. In contrast, passive micromixers that enhance mixing by modifying the microchannel geometry, are being widely developed due to the advantages of simple fabrication and easy integration with the microfluidic systems [3,5,6,7,8].

Over the last two decades, many researchers have proposed different microchannel designs to enhance the mixing performance of passive micromixers. In general, passive micromixers, depending upon the mechanism of mixing, are classified as lamination-based [9,10,11,12,13] or chaotic advection-based [14,15,16,17]. Kim et al. [9] proposed a serpentine laminating micromixer that combines the mixing mechanisms of split-and-recombination (SAR) using successive arrangement of F-shaped mixing units in two layers and chaotic advection through a three-dimensional (3D) serpentine microchannel path. Tofteberg et al. [10] developed a lamination micromixer incorporating a sequence of SAR process with patterning of the channel bottom with grooves. Nimafar et al. [12] proposed an H-micromixer based on SAR process, and compared its mixing performance with those of T- and O-micromixer in a Reynolds number range of 0.08–4.16. The results showed that the H-micromixer achieved 98% mixing at *Re* = 0.083 due to SAR process, which was much higher than those of the other two micromixers.

Liu et al. [14] proposed a 3D serpentine micromixer with C-shaped repeating units, and experimentally demonstrated its high mixing performance for Reynolds numbers larger than 25. Stroock et al. [15] proposed a micromixer with patterned grooves on the channel bottom to induce chaotic mixing at low Reynolds numbers. Xia et al. [16] proposed two chaotic micromixers consisting of double-layer crossing channels. Their numerical and experimental results demonstrated that chaotic advection that is generated through continuous stretching and folding along with splitting and recombination, even at low Reynolds numbers (*Re* < 0.2), resulted in high mixing performance. The et al. [18] proposed a shifted trapezoidal blade micromixer that combined several mixing principles, i.e., vortices, transversal flows, and chaotic advection, to attain a stable mixing efficiency of larger than 80% in a wide Reynolds number range of 0.5–100.

Numerical optimization techniques coupled with computational fluid dynamics (CFD) analysis based on 3D Navier-Stokes equations have been widely used as an efficient tool for design of micromixers [19,20,21,22,23]. Ansari and Kim [19,20] optimized geometric parameters of a staggered herringbone groove micromixer (SHM) with grooves at the bottom wall by using radial basis neural network (RBNN) and response surface approximation (RSA) as surrogate models. These optimizations were carried out using mixing index as a single objective function with two or three design variables. Kim et al. [21] performed an optimization of microscale vortex generators in a micromixer using an advanced RSA by considering four geometric design variables. It was found that a mixing uniformity of larger than 95% was obtained within a channel length of 1344 µm with an optimized microchannel configuration. Cortes-Quiroz et al. [22] applied a multi-objective genetic algorithm (MOGA) for shape optimization of a SHM at *Re* = 1 and 10, by integrating CFD calculations with a surrogate model. In this optimization, a Pareto-optimal front of trade-offs between two objective functions, i.e., mixing index at the exit and pressure drop, was generated by MOGA. Afzal and Kim [23] performed a multi-objective optimization of a SHM with three objective functions, i.e., mixing index at the exit, friction factor, and mixing sensitivity. Hossain and Kim [24] optimized a micromixer with two-layer serpentine crossing channels at Reynolds numbers of 0.2 and 40 by using CFD, surrogate model and MOGA. Kriging metamodel was used as a surrogate model, while mixing index at the exit and pressure drop were used as two objective functions to generate Pareto-optimal front.

In a recent study, Raza et al. [25] proposed a 3D serpentine SAR micromixer with OX-shaped mixing units, and analyzed the flow structure and mixing performance numerically in a wide Reynolds number range of 0.1–200. The proposed micromixer with five mixing units showed excellent mixing performance over the entire range of Reynolds number, through stretching and folding of fluid interface at the crossing channel intersection nodes at low Reynolds numbers and chaotic motion due to the 3D serpentine path at high Reynolds numbers. Especially, the enhancement of mixing performance in low-Re range is noticeable as compared to previous micromixers.

In the present work, multi-objective optimizations have been performed to further enhance the performance of the micromixer proposed by Raza et al. [25], at both low and high Reynolds numbers. Mixing index at the exit of the micromixer and pressure drop though the micromixer were employed as two objective functions. The Pareto-optimal fronts compromising these two objectives were generated using RBNN surrogate model and MOGA. The optimizations were carried out with the two objective functions at the two different Reynolds numbers, *Re* = 1 and 120. The corresponding Peclet numbers are 1 × 10^4^ and 120 × 10^4^, respectively.

## 2. Micromixer Geometry 

**Figure 1:**
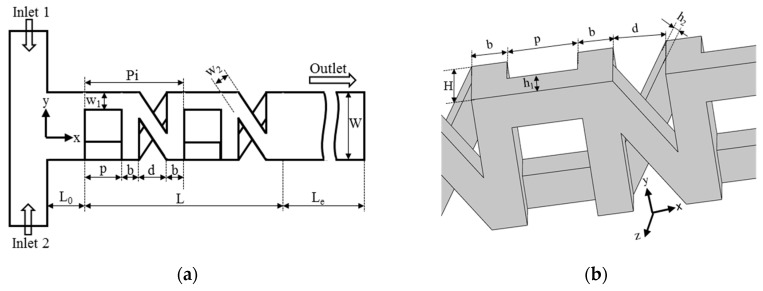
Geometry of micromixer with OX- shaped units: (**a**) planar view [25]; and, (**b**) three-dimensional (3D) view.

The micromixer configuration, as proposed by Raza et al. [25], has been used for the optimization. The micromixer consists of two repeated OX-shaped mixing units of 3D serpentine SAR structure as shown in Figure 1. The number of mixing units was reduced from five of previous work [25] to two in this work. Chaotic advection through stretching and folding of fluid interface at the X-junction, where the fluids in both the crossing channels are exchanged, enhances mixing at low Reynolds numbers. On the other hand, 3D serpentine path that was created by O-structure promotes mixing by producing chaotic advection at high Reynolds numbers, as explained in the previous study [25].

Values of the geometric parameters for the reference micromixer are listed in Table 1. The traditional method of stacking the polydimethylsiloxane (PDMS) layers can be used to fabricate the proposed micromixer. As described in the previous works [17,26], the micro-molding technology, i.e., soft lithography technique using SU-8 master molds, can be employed to fabricate these PDMS layers.

## 3. Numerical Analysis

The analyses of fluid flow and mixing inside the micromixer were performed numerically using a commercial CFD code, ANSYS CFX 15.0 (ANSYS, Inc., Canonsburg, PA, USA) [27], based on finite volume approximations. The numerical analysis of steady, incompressible, 3D laminar flow was carried out by solving the continuity and Navier-Stokes equations:

(1) $$\nabla \cdot \vec{V}=0$$

(2) $$\left(\vec{V}\cdot \nabla \right)\vec{V}=-\frac{1}{\rho }\nabla P+\nu \nabla^{2}\vec{V}$$

where the symbols *ν*, *ρ*, and $\;\vec{V}\;$ denote the fluid kinematic viscosity, density, and velocity, respectively. A solution of dye in water and water at a temperature of 20 °C were considered as two working fluids. To model the mass transport of the fluids having constant viscosity and density in the mixing process, a scalar transport equation of advection-diffusion type [28] was used, as follows:

(3) $$\left(\vec{V}\cdot \nabla \right)C=\alpha \nabla^{2}C$$

where the symbols *α* and *C* denote the diffusivity coefficient and dye concentration, respectively. In order to model mixing of two fluids, the scalar transport equation has been used and validated previously for different micromixers [29,30].

The computational domain was discretized by a combination of tetrahedral and hexahedral elements to reduce the total number of computational nodes. The mixing units were meshed with tetrahedral element due to their complex geometry, while the remaining part in the microchannel was meshed with hexahedral elements.

Uniform velocity profiles were assigned at the inlets, while the atmospheric pressure was used at the outlet. At the walls, no-slip condition was used. A solution of dye in water (mass fraction equal to 1) and water (mass fraction equal to 0) were introduced at the inlet 1 and inlet 2, respectively. The diffusivity coefficient value of the water-dye mixture was 1.0 × 10^−10^ m^2^/s. The values of density and dynamic viscosity of water (and also water-dye mixture) were 1000 kg/m^3^ and 1.0 × 10^−3^ kg·m^−1^·s^−1^, respectively [31]. Truncation errors that were associated with numerical discretization for the advection terms in the governing differential equations give rise to numerical diffusion. The extent of numerical diffusion depends on the numerical scheme used. Numerical diffusion can be decreased by using higher order approximation schemes, such as second-order upwind and third order QUICK [32] scheme instead of first-order upwind scheme [33]. A high-resolution scheme of second-order approximation was applied for discretization of advection terms. The solution convergence criterion was set as root-mean-square residual value of less than 1.0 × 10^−6^.

A statistical method [34] based on concept of intensity of segregation was used to define mixing index. The mixing index at a plane perpendicular to streamwise direction is represented as follows:

(4) $$M=1-\frac{\sigma }{\sigma_{max}}$$

where *σ* and $\sigma_{max}$ are the standard deviation of the concentration at the cross-sectional plane, and maximum standard deviation over the entire data range, respectively.

The standard deviation of dye mass fraction at the cross-sectional plane can be written as:

(5) $$\sigma =\sqrt{\frac{1}{N}\overset{N}{\underset{i=1}{\sum }}{\left(c_{i}-{\bar{c}}_{m\;}\right)}^{2}}$$

where *c_i_*, ${\bar{c}}_{m}$, and *N* are the mass fraction at sampling point *i*, the optimal mixing mass fraction, and the number of sampling points on the plane, respectively. A mixing index value of 0 indicates completely unmixed fluids, while a value of 1 indicates completely mixed fluids. Mixing index at the exit (*M_o_*) was defined as the mixing index 700 µm downstream of the main channel starting position.

The Reynolds Number was defined using hydraulic diameter (*D_h_*) of the main channel as follows:

(6) $$Re=\frac{\rho VD_{h}}{\mu }$$

where *ρ*, *µ*, and *V* denote the density of water, dynamic viscosity of water, and fluid average velocity, respectively.

Hydraulic diameter was defined as follows:

(7) $$D_{h}=\frac{2\times W\times H}{\left(W+H\right)}$$

where *W* and *H* denote the width and depth of the microchannel.

## 4. Design Variables and Objective Functions

**Figure 2:**
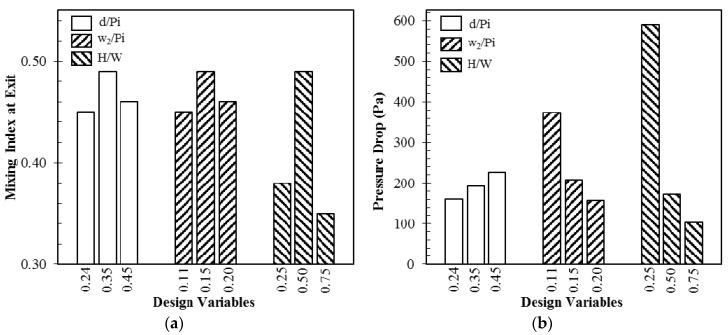
Effects of design variables on objective functions at *Re* = 1: (**a**) mixing index at the exit; and, (**b**) pressure drop.

For the optimization, the ratios of distance between O-structures to pitch (*d*/*Pi*), crossing channel width to pitch (*w*_2_/*Pi*), and total depth of the micromixer to main channel width (*H*/*W*), as shown in Figure 1, were selected as design variables among various geometric parameters through a preliminary parametric study. Effects of these design variables on mixing index at the exit and pressure drop through the micromixer at *Re* = 1 for the reference micromixer, are shown in Figure 2. For all of the design variables, the mixing index shows maximum values in the tested ranges of the variables. Pressure drop increases with increase in *d*/*Pi*, while it decreases with increase in *w*_2_/*P*i and *H*/*W* in the tested ranges. Design ranges for the design variables are summarized in Table 2.

In this work, mixing index at the exit and pressure drop in the micromixer were used to define two objective functions. The pressure drop that affects the power required to drive the fluids through the micromixer, was calculated as the difference in area-weighted average of the total pressure between the planes at the main channel inlet and exit. To make the optimization problem be defined as minimization of the objective functions, one of the objective functions (*F_M_*) was taken as the negative of mixing index, and the other objective function (*F*_∆*P*_) was defined as pressure drop. Two pairs of objective functions were selected as follows: mixing index at *Re* = 1 (*F_M_*_at *Re* = 1_)—pressure drop at *Re* = 1 (*F*_∆*P* at *Re* = 1_) and mixing index at *Re* = 120 (*F_M_*_at *Re* = 120_)—pressure drop at *Re* = 120 (*F*_∆*P* at *Re* = 120_). Values of the objective functions for the reference design are listed in Table 3.

## 5. Surrogate Model and Multi-Objective Optimization

**Figure 3:**
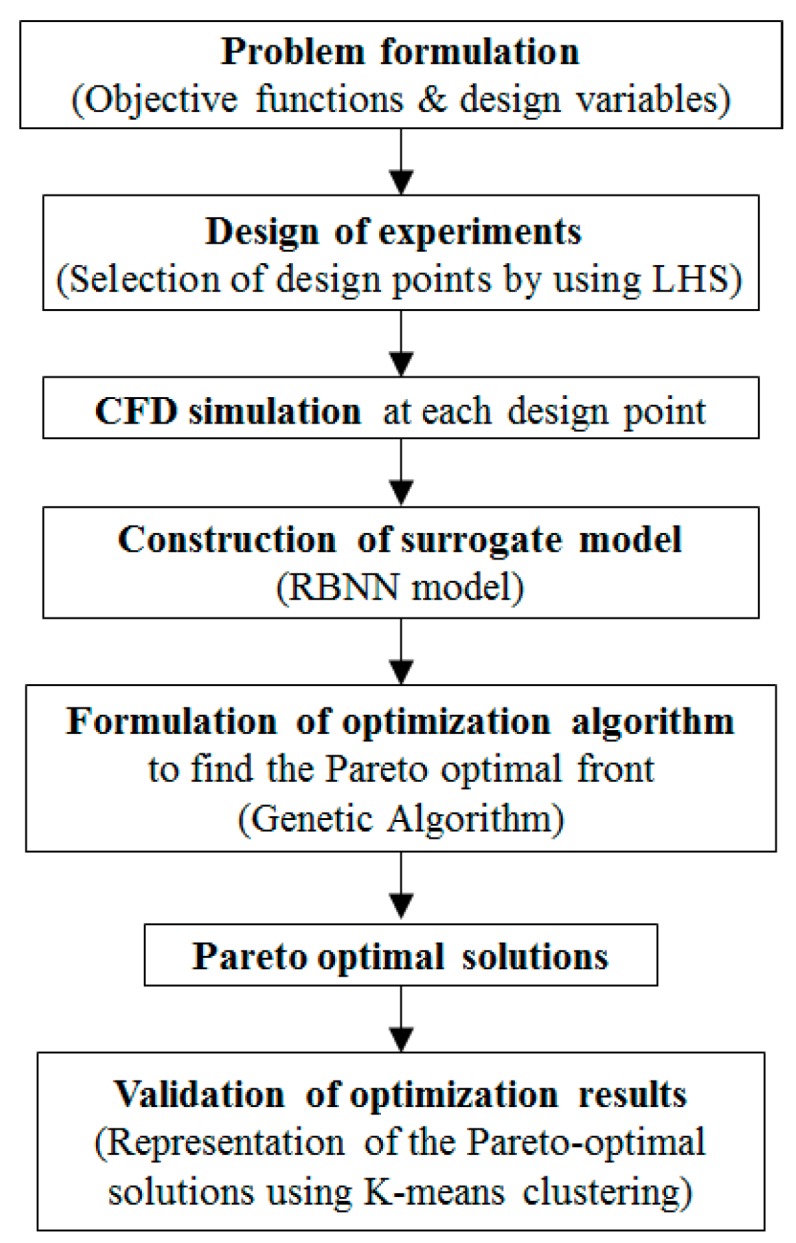
Multi-objective optimization procedure.

Procedure of multi-objective optimization used in the present work is represented in Figure 3. The first step is selecting design variables and their ranges through a previous parametric study, and the objective functions considering the design goals. The next step is to build the design space using the design of experiments (DOE). In the present work, Latin Hypercube Sampling (LHS) [35] is used as DOE. LHS is an effective sampling technique that uses an *m × n* simulation matrix where *m* is the number of levels (sampling points) to be examined and *n* is the number of design parameters. Each of the *n* columns of the matrix containing the levels, 1, 2, … , *m*, is randomly paired to form a Latin hypercube. This approach produces random sample points, ensuring that all of the portions of the design space are represented. Objective function values are calculated by Navier-Stokes analysis at these design points. A surrogate model is constructed to approximate the objective functions based on these objective function values, and MOGA is used to explore global Pareto-optimal solutions in the design space. If the global optimum solution exists in the design space and a termination criterion is satisfied, the multi-objective optimization procedure terminates.

The multi objective optimization problem was formulated, as follows:

Minimization: ***F***(***x***) = [*F*_1_(***x***), *F*_2_(***x***), *F*_3_(***x***)… *F_n_*(***x***)]

Design variable bound: ***LB*** ≤ ***x*** ≤ ***UB***, ***x***∈ ***R***

where ***F*(*x*)** is an objective function, **x** is a vector of design variables, and ***LB*** and **UB** denote the vectors of the lower and upper bounds, respectively.

In order to obtain LHS design points, MATLAB (MathWorks, Inc., Natick, MA, USA) function ‘*lhsdesign*’ [36] was used with the criterion ‘*maxmin*’ (maximize minimum distance between adjacent design point). As a result, uniformly distributed 27 design points were selected for the three design variables in order to construct a surrogate model. The objective function values calculated at these design points by Navier-Stokes analysis are listed in Table 4.

**Figure 4:**
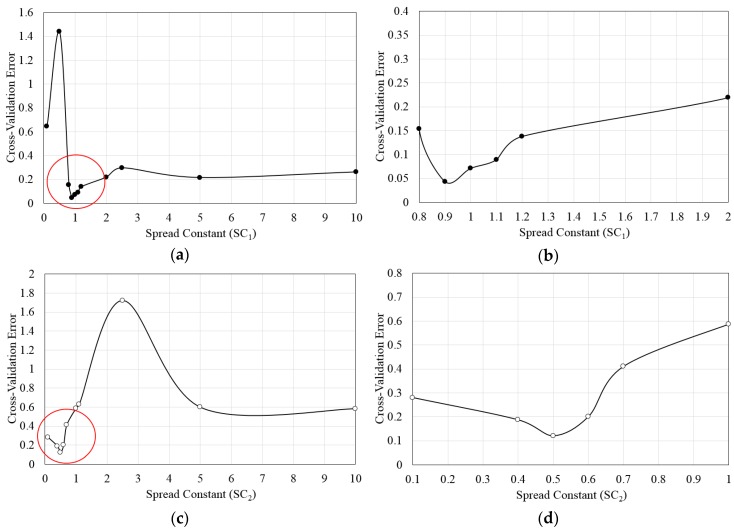
Cross-validation error vs. spread constant (*SC*) for the objective functions: (**a**) Mixing index (*SC*_1_); (**b**) Zoomed in view of encircled area in Figure 4a; (**c**) Pressure drop (*SC*_2_); (**d**) Zoomed in view of encircled area in Figure 4c.

A RBNN model [37] was used to construct surrogate models of the objective functions. The RBNN model contains a two-layered network comprised of a hidden layer of radial neuron and an output layer of linear neuron. The hidden layer executes a nonlinear alteration of the input space to a middle space by using a set of radial basis elements. The output layer then implements a linear combiner to yield the desired targets. The linear model *g*(*x*) for the function can be expressed as a linear combination of a set of *M* radially-symmetric functions, as follows:

(8) $$g\left(x\right)=\overset{M}{\underset{j=1}{\sum }}w_{j}\Phi_{j}$$

where *w_j_* is weights and *Φ_j_* are radial basis functions. Benefit of this surrogate modeling is an ability to reduce computational time owing to the linear nature of the radial basis functions. In the present work, the RBNN function, *newrb* [36] was used to construct the models. The network training was performed by varying spread constant (*SC*) to adjust the cross-validation error. In this study, *SC*_1_ is related to mixing index, and *SC*_2_ is related to pressure drop. The *SC* values of each objective function were selected through a k-fold cross-validation [38] error test in which the errors were minimum at *SC*_1_ = 0.9 and *SC*_2_ = 0.5, as shown in Figure 4. The k-fold cross-validation is a validation method to estimate how the results of a statistical analysis generalize to an independent data set.

MOGA was used to find optimal solution on the constructed surrogate model using the MATLAB optimization tool box [36]. MOGA is a randomized global search method that solves functions by imitating progressions observed from natural evolution [37]. Based on the survival and growth of the fittest, MOGA finds new and improved results repeatedly. MOGA describes an initial population of individuals, which represent a part of the solution to the functions. Before the search begins, a series of chromosomes is randomly selected from the design space to obtain the initial population. Through computations, the individuals are selected in a competitive way, based on their fitness functions as measured by each specific objective function. The genetic search operators (i.e., “selection”, “mutation”, and “crossover”) were applied to obtain a next generation of chromosomes, for which the predicted quality of all the chromosomes is better than that of the previous generation. This process is repeated until the termination criterion, which is function tolerance, is met. The following parameters were used: population size = 400, cross over fraction = 0.7, generations = 800, and function tolerance =10^−8^.

## 6. Results and Discussion

**Figure 5:**
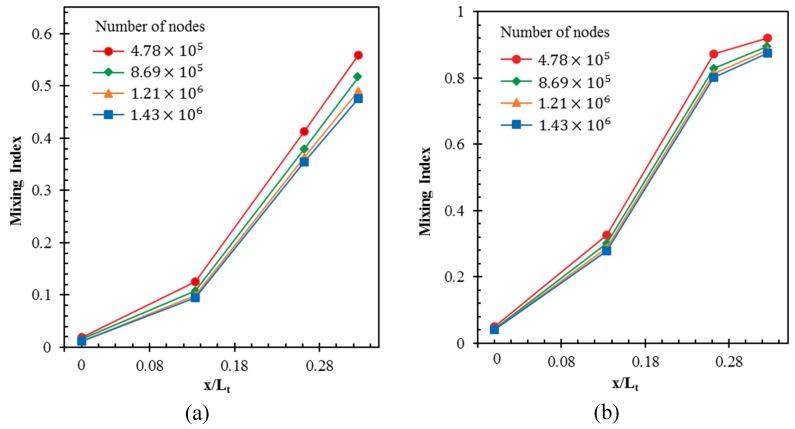
Grid-dependency tests for mixing index development along axial length of the reference design: (**a**) *Re* = 1; and, (**b**) *Re* = 120.

A grid-dependency test was performed to find out an optimal number of nodes for the spatial discretization of computational space. Four different grid systems with 4.78 × 10^5^ to 1.43 × 10^6^ nodes were tested for development of mixing index along the channel length at two different Reynolds numbers (*Re* = 1 and 120), as shown in Figure 5. The mixing indices were calculated on the planes perpendicular to the axial direction at four different locations (i.e., start of the main channel, two successive intersection nodes of the crossing channels in X-structure, and the exit). Almost similar profiles of the mixing index development are observed for grid systems with 1.21 × 10^6^ and 1.43 × 10^6^ nodes at both *Re* = 1 and 120. Hence, grid system with 1.21 × 10^6^ nodes was selected as an optimal grid, commonly at *Re* = 1 and 120.

**Figure 6:**
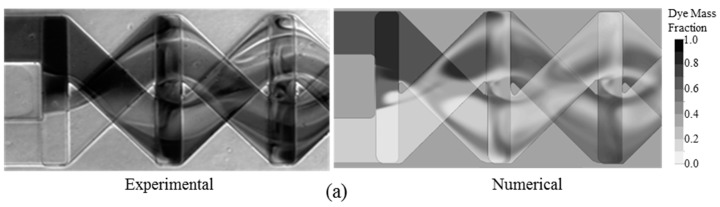

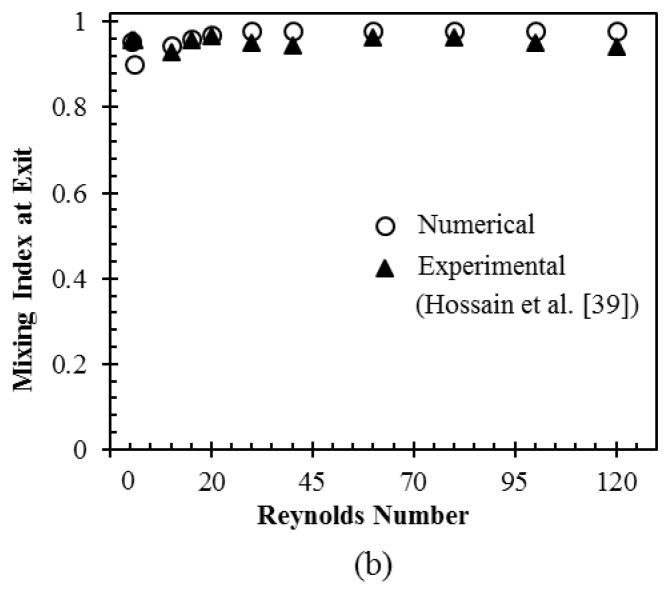
Validation of numerical results compared with experimental data [25]: (**a**) qualitative comparison of dye mass fraction distribution on *x-y* plane located halfway along the channel depth between experiment and numerical analysis at *Re* = 60; and, (**b**) quantitative comparison of mixing indices at the exit for different Reynolds numbers between experiment and numerical analysis.

The present numerical model was validated in the previous study [25] by both qualitative and quantitative comparisons of the numerical results with the experimental results of Hossain et al. [39], as shown in Figure 6. The optical image of fluid mixing in the first mixing unit at *Re* = 60 is compared with the numerical result of dye mass fraction distribution on *x*-*y* plane located halfway along the channel depth, as shown in Figure 6a. The numerical values of mixing indices at the exit of the micromixer in a Reynolds number range of 0.2–120 are also compared with the experimental data, as shown in Figure 6b. The numerically predicted mixing indices are slightly higher than the experimental data over the whole range. The uncertainties that are involved in the experimental procedures, such as capturing and analyzing experimental image, geometrical variation in fabrication, and wall roughness, can be attributed as the causes for the differences in the mixing indices, as shown in Figure 6b. However, the qualitative and quantitative comparisons between the numerical and experimental results, show acceptable agreements.

**Figure 7:**
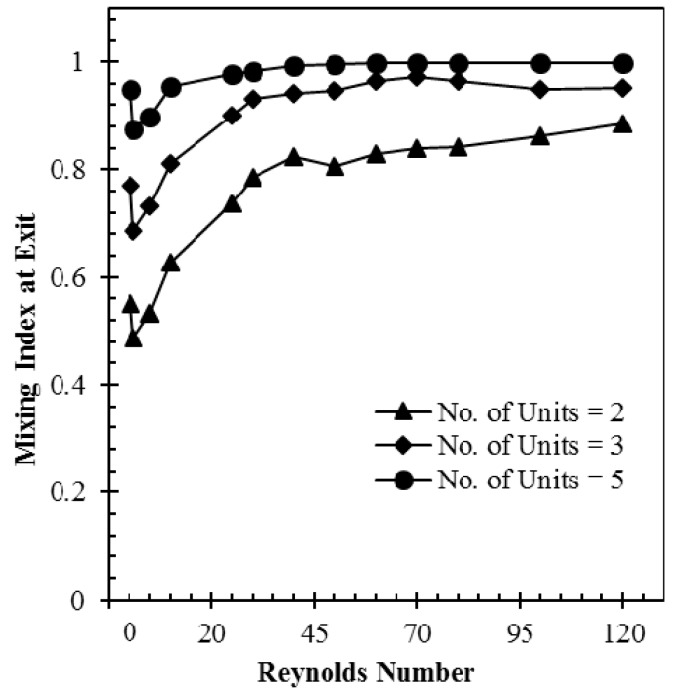
Variations of mixing index at the exit with Reynolds number for three different numbers of mixing units.

Figure 7 shows effect of number of the mixing units on the mixing performance through a quantitative comparison among the mixing indices at the exits of the micromixers with two, three, and five mixing units at different Reynolds numbers. It is observed that the micromixer with five mixing units exhibits almost complete mixing in most of the Reynolds number range, while the micromixers with two and three mixing units show largely reduced mixing index values over the whole *Re* range. It is also observed that all the micromixers show minimum mixing indices at *Re* = 1. Hence, the micromixer with two mixing units was selected in the present work to have more space for enhancement of the mixing performance by optimization.

**Figure 8:**
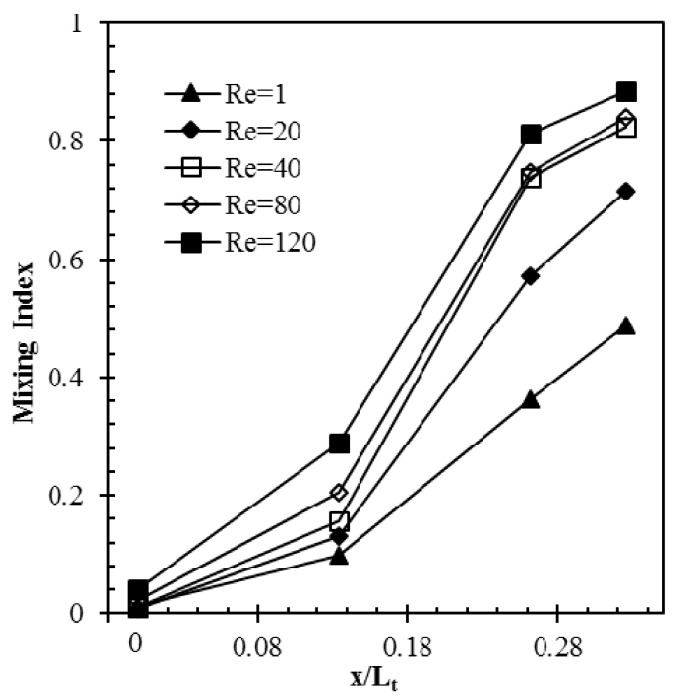
Developments of mixing index along the axial length of micromixer (reference design) for different Reynolds numbers.

Effect of Reynolds number on development of mixing index along the axial length of the micromixer (reference design) is shown in Figure 8. The mixing indices were calculated on four *y*-*z* planes (at the start of the main channel, the two successive intersection nodes of the crossing channels in X-structure, and the exit) for Reynolds numbers, 1, 20, 40, 80, and 120. The results show that developing rate of mixing index depends on Reynolds number, as shown in Figure 8. The rate of development of mixing index is found to be highest between the first and second intersection nodes at *Re* = 40.

**Figure 9:**
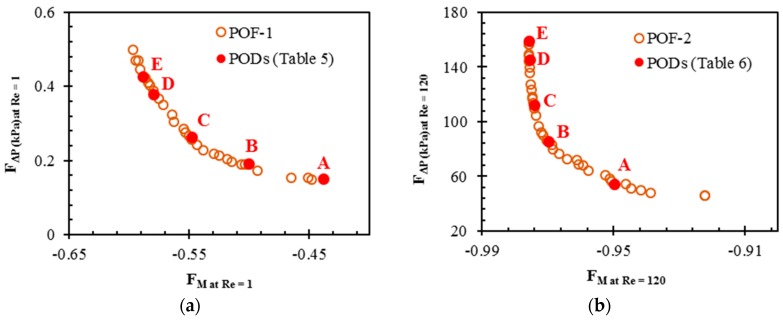
Pareto-optimal fronts: (**a**) mixing index at *Re* = 1 vs. pressure drop at *Re* = 1; and (**b**) mixing index at *Re* = 120 vs. pressure drop at *Re* = 120.

Following the procedure outlined in Figure 3, Pareto-optimal fronts presenting the optimum trade-off between the two conflicting objectives are plotted, as shown in Figure 9. Pareto-optimal fronts, termed as POF-1 and POF-2 in the Figure 9a,b, respectively, represent two pairs of the objective functions at two different Reynolds numbers: *F_M_*_at *Re* = 1_ − *F*_∆*P* at *Re* = 1_ and *F_M_*_at *Re* = 120_ − *F*_∆*P* at *Re* = 120_. Concave shape of the Pareto-optimal fronts indicates that an improvement in mixing index occurs with simultaneous increase in pressure drop. Selection of Pareto-optimal solution depends upon the choice of the designer, since each solution is a global Pareto-optimal solution and none of these Pareto-optimal solutions is superior to the others for both objective functions.

In order to analyze the Pareto-optimal solutions, five representative Pareto-optimal designs (PODs) were selected by K-means clustering on each Pareto-optimal front, as shown in Figure 9. PODs A and E at extreme ends of each Pareto-optimal front represent pressure drop-oriented and mixing index-oriented designs, respectively. Accomplishment of one objective function leads to forfeit of the other objective function. At *Re* = 1, as compared to POD A on POF-1, the mixing index-oriented design, POD E shows a relative enhancement of 34.5 % in the mixing index at the exit. The pressure drop-oriented design, POD A shows a 64.5 % reduction in pressure drop as compared to POD E. Similarly, at *Re* = 120, POD E shows a relative enhancement of 2.8 % in mixing index at the exit as compared to POD A on POF-2, and POD A shows a 65.7% reduction in pressure drop as compared to POD E. These results reveal that the relative enhancement of mixing index at the exit is much more pronounced at *Re* = 1 as compared to *Re* = 120. However, the relative percentage reductions in pressure drop are not much different at both the Reynolds numbers. The higher relative percentage changes in pressure drop than those in mixing index at the exit indicate that mixing index is less sensitive to the design variables as compared to pressure drop.

The results at the representative PODs for POF-1 and POF-2 are listed in Table 5 and Table 6, respectively. It is observed that mixing index at the exit and pressure drop are most sensitive to the design variable, *H*/*W*, while *w*_2_/*Pi* remains nearly invariant on POF-1 with the exception of POD A. A high value of mixing index is observed for *d*/*Pi*, *w*_2_/*Pi*, and *H*/*W* values close to middle of the range, upper bound, and lower bound, respectively. In case of POF-2, the objective functions become more sensitive to the design variables when compared to POF-1. The results of numerical analysis at the PODs shows that maximum enhancements of 18.8% (POD E on POF-1) and 6.0% (POD E on POF-2) in mixing index at the exit are achieved as compared to the reference design at *Re* = 1 and 120, respectively, by the optimization. Maximum reductions of 5.8% (POD A on POF-1) and 11.1% (POD A on POF-2) in pressure drop are obtained as compared to the reference design at *Re* = 1 and 120, respectively. The surrogate model predictions of the objective functions values are also compared with the numerical results at the same designs in Table 5 and Table 6. The relative errors of the surrogate predictions for mixing index at the exit and pressure drop are less than 2% and 10%, respectively, at *Re* = 1, as shown in Table 5. However, these relative errors are increased at *Re* = 120 to less than 5% and 22%, respectively, as shown in Table 6.

**Figure 10:**
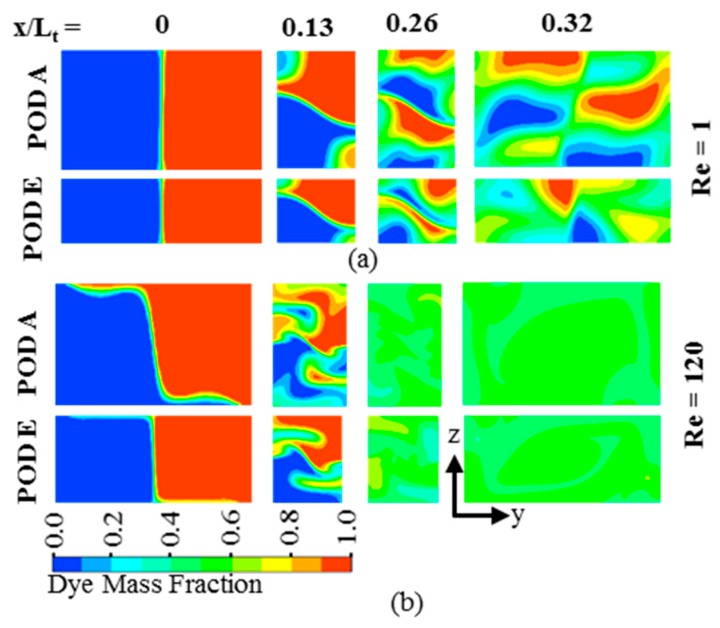
Dye mass fraction distributions on *y*-*z* planes for POD A and POD E: (**a**) Pareto-optimal fronts (POF)-1 (*Re* = 1); and (**b**) POF-2 (*Re* = 120).

For qualitative comparison of mixings at different PODs on POF-1 (*Re* = 1) and POF-2 (*Re* = 120), dye mass fraction contours are plotted on *y*-*z* planes at the beginning of the main channel (*x*/*L_t_* = 0), two successive intersection nodes of the crossing channel in X-structure (*x*/*L_t_* = 0.13 and 0.26), and the exit (*x*/*L_t_* = 0.32), respectively, as shown in Figure 10. Two Pareto-optimal designs, i.e. POD A and POD E at the extreme ends of each Pareto-optimal front are selected for the comparison. Number of two-fluid interfaces increases along the channel length for both the PODs. However, there is a distinct difference in the dye mass fraction distribution at the exit of the micromixer between POD A and POD E on POF-1. A more uniform dye mass fraction distribution is observed at the exit for POD E as compared to POD A on POF-1, as shown in Figure 10a, whereas almost similar distributions are observed for POD A and POD E on POF-2 (Figure 10b). This is related to the fact that the change in mixing index along POF-1 (0.439–0.581 in Table 5) is much larger than that along POF-2 (0.936–0.957 in Table 6).

**Figure 11:**
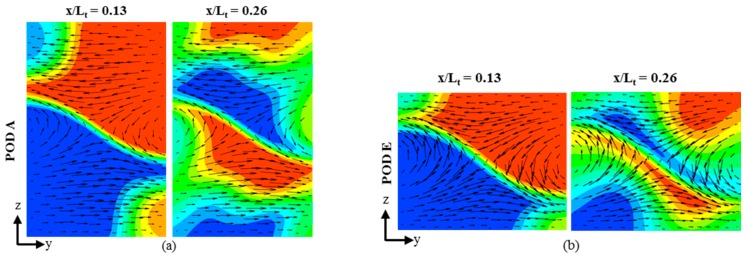
Velocity vectors and concentration contours on *y*-*z* planes at first (*x*/*L_t_* = 0.13) and second (*x*/*L_t_*=0.26) X-structure intersection nodes: (**a**) POD A; and, (**b**) POD E, on POF-1.

In order to analyze the mixing mechanism, velocity vectors superimposed on the dye mass fraction contours are plotted for POD A and POD E on POF-1 (*Re* = 1), in Figure 11. It is observed that a saddle-shaped flow structure, which promotes chaotic advection [25], is formed at each intersection node (*x*/*L_t_* = 0.13 and 0.26) of the crossing microchannels in both of the Pareto-optimal designs, POD A and POD E. However, there is a difference in the fraction of total depth of the microchannel cross-section covered by saddle-shaped pattern between the two designs. In case of POD A, the saddle-shaped flow structure exists only in the middle height of the *y*-*z* plane, as shown in Figure 11a. Whereas, in case of POD E, the saddle-shaped flow pattern covers more height of the *y*-*z* plane, as shown in Figure 11b. This results in more uniform mass fraction distribution (Figure 11b) and consequently, higher mixing index in POD E, as compared to POD A.

**Figure 12:**
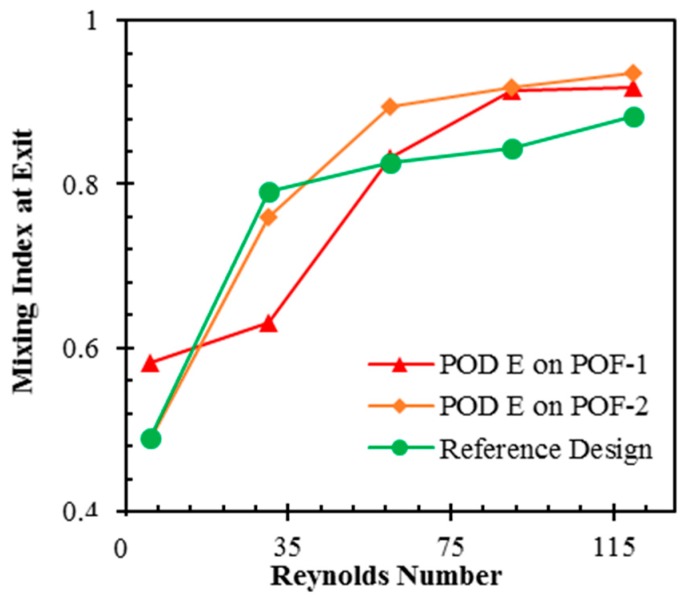
Variations of mixing index at the exit with Reynolds number for POD E on POF-1, POD E on POF-2, and reference design.

In order to have an idea about the mixing performance of a Pareto-optimal design at Reynolds numbers other than the Reynolds number at which it was obtained, mixing performances of PODs E on POF-1 and POF-2 are compared with the reference design at Reynolds numbers, 1, 30, 60, 90, and 120, as shown in Figure 12. It is observed that POD E on POF-1 shows values of mixing index larger than those of the reference design except at intermediate Reynolds number of 30, but the mixing index values are smaller than those of POD E on POF-2 except at *Re* = 1. POD E on POF-2 shows improvements in the mixing index at Reynolds numbers larger than 60 when compared to the other designs.

In order to compare the merit of PODs at *Re* = 1 with previous micromixers, as shown in Table 7, two different mixing performance parameters named, “mixing cost” [40], and “mixing energy cost” (*mec*) [41] have been used. The terms, “mixing cost” and “mixing energy cost” are defined as:

(9) $$mixing\;cost=\frac{\eta }{\Delta P}$$

(10) $$mec=\frac{{\bar{C}}_{p}}{\eta }$$

where *η*, Δ*P*, and ${\bar{C}}_{p}$ denote mixing efficiency, pressure drop and mean input power coefficient, respectively. The detail description about these terms can be found in the works of Ortega-Casanova [41,42]. Mixing efficiency (*η*) and mean input power coefficient (${\bar{C}}_{p}$) are defined as follows:

(11) $$\eta =\left(1-\frac{\sigma }{\sigma_{max}}\right)\times 100$$

(12) $${\bar{C}}_{p}=2\Delta Pq/\rho V^{2}$$

where *σ*, $\sigma_{max}$, Δ*P*, *q*, *ρ*, and *V* are the standard deviation of the concentration, maximum standard deviation, pressure drop, dimensionless flow rate, density of fluid, and average flow velocity, respectively. A micromixer with higher mixing cost and lower *mec* indicates a more efficient micromixer. From Table 7, it is evident that PODs have neither the best nor the worst values of these parameters. Higher mixing cost values are shown by all of the PODs as compared to the micromixer proposed by Cheri et al. [43]. PODs show higher *mec* values as compared to the rhombic micromixer with asymmetrical flow [40]. The highest *mec* value is shown by the micromixer configuration of a rectangular chamber with obstruction proposed by Cheri et al. [43]. Although the micromixer proposed by Cheri et al. [43] shows the highest pressure drop, due to the highest mixing efficiency and the lowest mean input power coefficient, the *mec* value becomes least.

## 7. Conclusions

Multi-objective optimizations of a micromixer with 3D serpentine and SAR configuration have been performed at Reynolds numbers, 1 and 120, based on flow and mixing analyses using 3D Navier-Stokes equations and convection-diffusion equation. Three design variables, i.e., *d*/*Pi*, *w*_2_/*Pi*, and *H*/*W*, were selected, and two objective functions were defined in terms of mixing index at the exit of the micromixer and pressure drop through the micromixer for the optimization. In a parametric study, the mixing index shows maxima for all of the design variables, but the pressure drop shows monotonic variations for all the design variables in the tested ranges. Two concave Pareto-optimal fronts (POF-1 and POF-2) representing trade-off between the two objective functions at *Re* = 1 and 120, respectively, were obtained by RBNN surrogate model and MOGA. In applying RBNN model, it was found that the k-hold cross-validation errors for mixing index and the pressure drop were minimized with the spread constants, 0.9 and 0.5, respectively. On POF-1 (*Re* = 1), the preference of a mixing index-oriented design, POD E over a pressure drop-oriented design, POD A leads to 34.5% relative increase in mixing index at the exit, and the preference of POD A over POD E showed 64.5% reduction in pressure drop. On POF-2 (*Re* = 120), the preference of POD E over POD A leads to only 2.8% relative increase in mixing index at the exit, and the preference of POD A over POD E showed 65.7% reduction in pressure drop. These results indicate that mixing index is less sensitive to the design variables as compared to pressure drop, on the Pareto-optimal fronts. It was found from the numerical analysis at the PODs that the maximum enhancements of 18.8% at POD E on POF-1 and 6.0% at POD E on POF-2 in mixing index at the exit were obtained when compared to the reference design. And, maximum reductions of 5.8% at POD A on POF-1 and 11.1% at POD A on POF-2 in pressure drop were achieved compared to the reference design. Maximum relative error of the surrogate prediction compared to the numerical analysis was smaller for mixing index than pressure drop, and increased with the Reynolds number. In a range of *Re* = 1–120, POD E on POF-1 showed values of mixing index larger than those of the reference design except at *Re* = 30, but POD E on POF-2 showed higher mixing performance than the reference design, only for the Reynolds numbers larger than 60.

## Figures and Tables

**Table 1:** Geometric parameters and their values.

Geometric Parameter	Value (In µm)
Length of initial part of main channel, *L*_0_	100
Exit channel length, *L_e_*	1500
Total length, *L_t_*	2150
Pitch length, *Pi*	275
Width of main channel, *W*	200
Width of O-structure, *w*_1_	50
Width of X-structure, *w*_2_	50
Distance between O-structures, *d*	75
Depth of horizontal portion of O-structure, *h*_1_	50
Depth of X-structure, *h*_2_	50
Total depth of main channel, *H*	100

**Table 2:** Design variables and their ranges.

Design Variables	Lower Limit	Upper Limit
*d*/*Pi*	0.236	0.455
*w*_2_/*Pi*	0.109	0.200
*H*/*W*	0.250	0.750

**Table 3:** Reference design and its objective functions values.

Reference Design						
Design Variables			Objective Functions			
*d*/*Pi*	*w*_2_/*Pi*	*H*/*W*	Mixing Index at exit, *M*_0_		Pressure drop, Δ*P* (kPa)	
			*Re* = 1	*Re* = 120	*Re* = 1	*Re* = 120
0.272	0.182	0.500	0.489	0.883	0.171	69.741

**Table 4:** Design variables and objective function values at Latin hypercube sampling (LHS) design points.

Design Point	Design Variables			Objective Functions (*Re* = 1)		Objective Functions (*Re* = 120)	
	*d*/*Pi*	*w*_2_/*Pi*	*H*/*W*	Mixing Index, *M_o_*	Pressure Drop, ∆*P* (kPa)	Mixing Index, *M_o_*	Pressure Drop, ∆*P* (kPa)
1	0.377	0.162	0.635	0.410	0.962	0.171	61.976
2	0.418	0.197	0.673	0.400	0.926	0.136	48.443
3	0.369	0.172	0.442	0.504	0.962	0.254	91.358
4	0.288	0.169	0.423	0.539	0.929	0.248	98.528
5	0.442	0.141	0.577	0.404	0.854	0.261	89.404
6	0.272	0.131	0.731	0.374	0.977	0.171	69.802
7	0.385	0.113	0.500	0.419	0.750	0.422	153.596
8	0.434	0.148	0.385	0.488	0.795	0.422	143.310
9	0.321	0.155	0.712	0.391	0.915	0.123	46.962
10	0.329	0.183	0.692	0.382	0.930	0.232	86.091
11	0.345	0.127	0.654	0.371	0.847	0.334	114.287
12	0.426	0.117	0.615	0.468	0.920	0.350	151.380
13	0.280	0.120	0.462	0.474	0.972	0.234	88.623
14	0.345	0.155	0.500	0.472	0.935	0.192	68.240
15	0.402	0.186	0.519	0.472	0.935	0.192	68.240
16	0.256	0.190	0.365	0.459	0.862	0.266	105.153
17	0.337	0.179	0.327	0.580	0.914	0.391	136.554
18	0.353	0.124	0.288	0.495	0.831	0.810	300.789
19	0.361	0.152	0.269	0.572	0.852	0.715	247.365
20	0.248	0.158	0.596	0.450	0.885	0.150	65.947
21	0.297	0.193	0.481	0.499	0.888	0.178	68.949
22	0.410	0.176	0.250	0.541	0.818	0.754	229.751
23	0.240	0.165	0.404	0.543	0.915	0.248	108.789
24	0.393	0.138	0.558	0.420	0.891	0.261	93.075
25	0.313	0.134	0.538	0.448	0.933	0.250	99.692
26	0.264	0.110	0.308	0.493	0.941	0.736	342.159
27	0.305	0.145	0.346	0.534	0.911	0.432	173.488

**Table 5:** Results of optimization at representative Pareto-optimal designs (PODs) for *Re* = 1.

Selected PODs	Design Variables			Surrogate Prediction		Numerical Analysis		% Error	
	*d*/*Pi*	*w*_2_/*Pi*	*H*/*W*	*M_o_*	Δ*P* (kPa)	*M_o_*	Δ*P* (kPa)	*M_o_*	Δ*P*
A	0.308	0.163	0.602	0.438	0.152	0.439	0.161	−0.23	−5.59
B	0.296	0.171	0.485	0.500	0.190	0.498	0.199	0.40	−4.52
C	0.318	0.175	0.404	0.548	0.265	0.551	0.269	−0.54	−1.49
D	0.327	0.175	0.338	0.580	0.378	0.587	0.373	−1.19	1.34
E	0.330	0.176	0.328	0.589	0.428	0.581	0.391	1.38	9.46

**Table 6:** Results of optimization at representative PODs for *Re* = 120.

Selected PODs	Design Variables			Surrogate Prediction		Numerical Analysis		% Error	
	*d*/*Pi*	*w*_2_/*Pi*	*H*/*W*	*M_o_*	Δ*P* (kPa)	*M_o_*	Δ*P* (kPa)	*M_o_*	Δ*P*
A	0.329	0.161	0.628	0.950	54.544	0.957	61.966	−0.73	−11.98
B	0.272	0.132	0.583	0.970	85.442	0.951	92.696	2.00	−7.83
C	0.254	0.123	0.472	0.974	112.168	0.936	142.565	4.06	−21.32
D	0.245	0.120	0.464	0.976	145.752	0.948	154.631	2.95	−5.74
E	0.241	0.120	0.446	0.976	158.992	0.936	166.768	4.27	−4.66

**Table 7:** Comparison of mixing cost and mixing energy cost of PODs on POF-1 with previous micromixers.

	Previous Works								
	PODs on POF-1					Ortega-Casanova [41]	Ortega-Casanova [42]	Chung and Shih [40]	Cheri et al. [43]
Parameters	A	B	C	D	E				
*η*	44	50	55	59	58	27	27	55	60
Δ*P* (Pa)	161	199	269	373	391	-*	-*	162	530
${\bar{C}}_{p}\;$	7273	6800	7141	7619	7624	904	550	10,000	100
*mixing cost*	0.273	0.251	0.205	0.157	0.149	-*	-*	0.3	0.113
*mec*	166	137	130	130	131	33	20	182	1.6

-* denotes data not available.

## Nomenclature

*b*	length of the vertical sub-channel in O-structure (m)
${\bar{C}}_{p}$	mean input power coefficient
*c*	mass fraction
*D_h_*	hydraulic diameter of the main channel (m)
*d*	spacing between two O-structure (m)
*F*	objective function
*H*	total depth of the channel (m)
*h*_1_	depth of the horizontal sub-channel in O-structure (m)
*h*_2_	depth of the sub-channel in X-structure (m)
*L_o_*	length of initial part of main channel (m)
*L*	total length of the mixing unit (m)
*L_e_*	length of the channel outlet (m)
*L_t_*	total length of the micromixer (m)
LHS	Latin hypercube sampling
*M*	mixing index
*M_o_*	mixing index at the exit
MOGA	multi-objective genetic algorithm
*N*	number of sampling points
*n*	number of mixing units
*P*	pressure (Pa)
Δ*P*	pressure drop (Pa)
*Pi*	pitch length (m)
POD	Pareto-optimal design
POF	Pareto-optimal front
*p*	length of the horizontal sub-channel in O-structure (m)
RBNN	radial basis neural network
*Re*	Reynolds number
SAR	split and recombine
*V*	average velocity (m/s)
*W*	width of the main channel (m)
*w*_1_	width of the sub-channel in O-structure (m)
*w*_2_	width of the sub-channel in X-structure (m)
*x*, *y*, *z*	Cartesian coordinates
**Greek Symbols**	
*α*	fluid diffusivity coefficient (m^2^/s)
*η*	mixing efficiency
*µ*	fluid dynamic viscosity (kg·m^−1^·s^−1^)
*ν*	fluid Kinematic viscosity (m^2^/s)
*ρ*	fluid density (kg/m^3^)
*σ*	standard deviation
**Subscripts**	
*i*	sampling point or fluid component
*m*	optimal mixing
*max*	maximum value
*x*	axial distance
